# Application of Combined Local and Global Optimization Algorithms in Joint Interpretation of Direct Current Resistivity and Seismic Refraction Data: A Case Study of Dammam Dome, Eastern Saudi Arabia

**DOI:** 10.3390/s22239337

**Published:** 2022-11-30

**Authors:** Paul Edigbue, Ismail Demirci, Irfan Akca, Hamdan Hamdan, Panagiotis Kirmizakis, Pantelis Soupios, Markos Tranos, Israa S. Abu-Mahfouz, Emin Candansayar, Sherif Hanafy, Abdullatif Al-Shuhail

**Affiliations:** 1 Department of Geosciences, College of Petroleum Engineering and Geosciences, King Fahd University of Petroleum and Minerals, Dhahran 31261, Saudi Arabia; 2 Department of Geophysical Engineering, Ankara University, Gölbaşı 06830, Turkey; 3 Department of Applied Physics & Astronomy, University of Sharjah, Sharjah 27272, United Arab Emirates; 4 Department of Geology, Aristotle University of Thessaloniki, 54124 Thessaloniki, Greece

**Keywords:** direct current resistivity, seismic refraction, local optimization, global optimization, joint inversion, Dammam Dome

## Abstract

The main geological structures in the Dammam Dome are defined by integrating geophysical measurements and applying new methodological approaches. Dammam Dome is characterized by a well-developed fracture/joints system; thus, high complexity of the subsurface is expected. Direct Current Resistivity (DCR) and Seismic Refraction (SR) geophysical survey aimed to map the Dammam Dome’s near-surface features. The geophysical data were acquired along two profiles in the northern part of Dammam Dome. To maximize the results from conducting DCR and SR measurements over a complex area, a combined local and global optimization algorithm was used to obtain high-resolution near-surface images in resistivity and velocity models. The local optimization technique involves individual and joint inversion of the DCR and SR data incorporating appropriate regularization parameters, while the global optimization uses single and multi-objective genetic algorithms in model parameter estimation. The combined algorithm uses the output from the local optimization method to define a search space for the global optimization algorithm. The results show that the local optimization produces satisfactory inverted models, and that the global optimization algorithm improves the local optimization results. The joint inversion and processing of the acquired data identified two major faults and a deformed zone with an almost N–S direction that corresponds with an outcrop were mapped in profile one, while profile two shows similar anomalies in both the resistivity and velocity models with the main E–W direction. This study not only demonstrates the capability of using the combined local and global optimization multi-objectives techniques to estimate model parameters of large datasets (i.e., 2D DCR and SR data), but also provides high-resolution subsurface images that can be used to study structural features of the Dammam Dome.

## 1. Introduction

Geophysical investigations are most effective in areas with sufficient subsurface geological contrasts, such as layer boundaries and lateral changes, including fractured/faulted or, in general, deformed zones [1]. These contrasts create a specific geophysical response in the field observations (geophysical measurements) and are interpreted with respect to the geological setting of the survey area. Conceptually, the method(s) applied in geophysical exploration depends on the physical properties, the contrast between the anomalies and the surroundings, and the location/geometry of expected anomalies [2]. The Dammam Dome, which has been related to salt and tectonic processes since Jurassic and Cretaceous times [3,4,5], offers an opportunity to apply both Seismic Refraction (SR) and Direct Current Resistivity (DCR) geophysical methods. It should be mentioned that resistivity (DCR) and seismic methods are suggested as the optimum methods for natural resources exploration, engineering, and environmental purposes [1]. This area has been chosen because extended earthworks are in progress for highway construction. As a result, an open cutting of the Dammam Dome was exposed, providing a unique opportunity to apply the aforementioned geophysical methods and thus get an insight into the stratigraphy and the well-developed fracture system of the Dammam Dome, as reported by previous studies [3,6,7,8,9].

The primary goal of inverting geophysical data is to generate a model that will give a theoretical response similar to the geophysical field measurements [10]. However, it is unusual to have a unique solution due to the ill-posed characteristics of the most geophysical inverse problem. To reduce uncertainties associated with the inversion of a dataset belonging to a single geophysical method, many researchers have applied the concept of local optimization methods and joint inversion [11,12,13,14,15,16,17] or data integration of more than one method, which provides better model resolutions than individual inversions [18,19,20,21,22]. For the local optimization method, the structural data coupling approach, which involves the cross-gradient constraint method, was applied [11]. The local optimization techniques are used iteratively to obtain an updated model that minimizes the objective function, which may not be the global solution of the inverse problem [23,24]. The global optimization algorithm in geophysical inversion is used to search for a solution space to avoid being trapped in the local minimum of the objective function [25,26,27,28,29,30]. Global optimization algorithms have been successfully applied to solve geophysical problems involving the gravity method [31,32], Magnetotelluric [33,34], and traveltime tomography [26]. Among the global optimization applicable to geophysical inversion, the genetic algorithm has been mostly applied because it does not involve complex computation and uses a crossover operator that enables information sharing among the models in the population [35].

Consequently, only the best solutions make it to the next generation. Some studies present integrated local and global optimization algorithms in hybrid form to extract the advantage of both optimization algorithms [25,26]. For this study, the genetic algorithms were used in two different forms. In the first one the genetic algorithm (GA) for a single objective function case was applied, and for the joint processing of the DCR and SR data the non-dominated sorting genetic algorithm 2 (NSGA II) for the multi-objective joint optimization approach was used. The use of genetic algorithms in a single objective function is a well-known and controlled problem [26], whereas one of the major challenges in applying the genetic algorithm or global optimization method to a multi-objective function (different geophysical datasets) is the determination of the optimum solutions that do not dominate each other [30].

This study presents the combined local and global optimization approach [36] to jointly invert SR and DCR data to improve the lateral and in-depth resolution and, thus, fully characterize the study area and delineate the presence of anomalous zones. In particular, we aim to outline probable near-surface features such as faults or fractures in the study area that can be correlated with the background geology (outcrop). This process would provide probable validation for the interpreted resistivity and velocity models. The unique aspect of this study is that it is the first time real DCR and SR datasets are used to test the feasibility of the combined optimization approach, utilizing single and multi-objective methods.

## 2. Materials and Methods

### 2.1. Geology of Study Area

**Figure 1:**
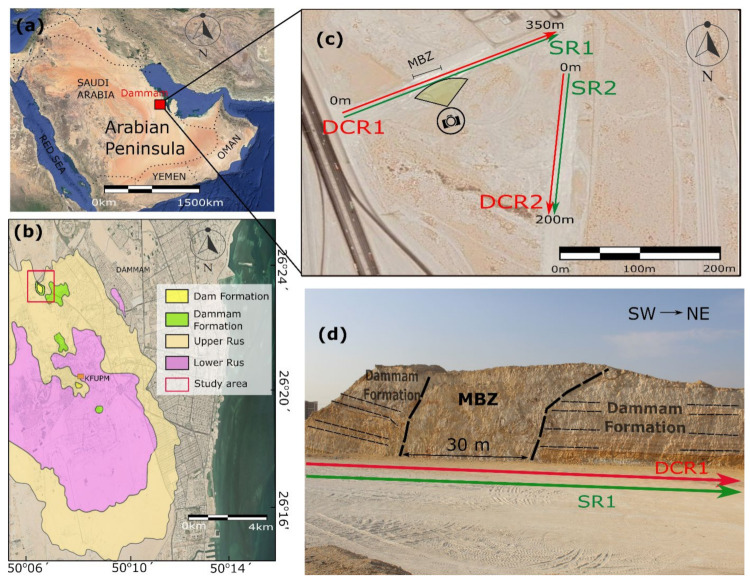
(**a**) Satellite image of the study area and (**b**) geological map of the wider study area (modified from Weijermars [4]). Red rectangle shows the study area; (**c**) Field view of the wide ‘megabreccia’ zone (MBZ) disrupting the sedimentary rocks of the Dammam Formation along the roadcut. The location from where the photo for figure (**c**) is taken, is shown. The DCR and SR profiles performed herein are shown with red (DCR1, DCR2) and green (SR1, SR2) lines; (**d**) The stratigraphy of the Dammam formation and the disruption (MBZ) zone of the sedimentary sequence are presented. The DCR1 (red arrow) and SR1 (green arrow) profiles collected along the under-construction road are depicted.

The study area is located on the right side of the road from Al Dhahran to Ad Dammam airport and about 2.7 km north of the King Abdulaziz Center of World Culture–Ithra crossroad, eastern Saudi Arabia (Figure 1a). The exposed sedimentary rocks in the area build up the northern part of the large oval-shaped dome [4]. The latter is the first, and main, hydrocarbon structural trap found in the Eastern Province of Saudi Arabia, and is widely known as the Dammam Dome [3]. More precisely, the Dammam Dome covers an area of ca. 500 km^2^ encompassing the cities of Al Khobar, Al Dhahran, and part of Ad Dammam [3]. Powers et al. [37] grouped the exposed sedimentary rocks of the Dammam Dome into the following formations from bottom to top: (a) Rus Formation; (b) Dammam Formation; (c) Hadrukh Formation; and (d) Dam Formation, with the first two formations dated in Eocene, whereas the next two upwards dated to Lower Miocene due to a major unconformity that is generally referred to as the Pre-Neogene Unconformity (PNU) [7] (Figure 1b). Based on the above, the Rus formation can be considered as the bedrock for our geophysical survey. Powers et al. [37] mapped in detail the Dammam Dome, whereas a geological compilation of the Dammam Peninsula from existing source maps [6,7,38] on a modern road map was compiled by Weijermars [4]. Hariri and Abdullatif [8], Al-Fahmi [9], Al-Fahmi et al. [39], and Hariri [3] have analyzed the fracture patterns of the Dammam Dome, which vary from place to place, but into which the NNW–SSE trending fractures form the regional fracture set. Based on the fracture patterns, Hariri and Abdullatif [8], Al-Fahmi [9], Al-Fahmi, et al. [39], and Hariri [3] concluded that the dome, as part of the interior of the Arabian platform, was far away from the Zagros Mountains belt and therefore was not influenced by the deformation processes taken part along the Zagros due to the convergence between the Arabian and Eurasia plates.

A more recent study by Tranos and Osman [5] dealing with the soft-sediment deformation in the area defined that the site in the Eocene was under a transtension stress regime associated with ENE–WSW compressive stresses, which originated along the Zagros mountain belt due to the collision between the two plates mentioned above. Such a transtensional stress regime was also defined by the orientations of the ENE–WSW striking, km-long, and almost vertical tectonic lines, which were found to cut the Dammam Dome after a geophysical exploration on the King Fahd University campus [40].

The site under investigation is a NE–SW road of ca. 500 m cutting across the sedimentary rocks of the Dammam Formation [7]. The Dammam Formation, coined by Bramkamp in 1941 (unpublished Saudi Aramco report; see Powers et al. [37]), rests conformably on top of the Rus Formation and is subdivided into five members [37]. From bottom to top, these are: (1) Midra Shale; (2) Saila Shale; (3) Alveolina Limestone; (4) Khobar limestone member; and (5) Alat dolomitic limestone/marl member. The three upper members are of Lutetian age (52.0 to 43.8 Ma) [41], whereas the two lower members are of Ypresian age (57.8 to 52.0 Ma) [41].

On the exposed NW slope roadcut (Figure 1c), the predominant feature is an NNW–SSE wide fault zone of ca. 30 m that disrupts the rocks of the Dammam Formation. In particular, the bedding seen clearly outside the fault zone cannot be traced within the fault zone due to the intense cataclasis and mingling, processes that make the fault zone appear like a ‘megabreccia’ zone (MBZ) (Figure 1d). As a result, the fault zone disrupts and mismatches the continuation of the bedding. This deformed zone has been recently interpreted by Alkhalifa and Kurison [42] as a karst collapse zone.

### 2.2. Geophysical Data Acquisition and Quality Control

Two DCR and SR profiles were conducted along road cuts, as shown in Figure 1. Profile 1 is 350 m long and addressed from southwest to northeast, while profile 2 is about 200 m long and acquired from almost north to south in the study area (Figure 1b). DCR data were acquired using the multi-electrode Syscal Pro Switch 96 resistivity instrument, using 75 electrodes with 5 m electrode spacing on profile 1, and 49 electrodes with the same spacing on profile 2. The dipole–dipole (DD) acquisition protocol was used in both DCR profiles 1 and 2 since the DD acquisition protocol provides the best lateral resolution [1], and based on the visual evidence mainly lateral changes were expected to be found. For the SR data, 120 shots and receivers at 3 m intervals were used on profile 1, while 40 shots and receivers at 5 m intervals were used on profile 2. The DCR data were converted to a format readable with our Matlab inversion algorithm that prepares the data for inversion. Data preparation for the SR method involves mainly the first arrival picking. The first arrival picking technique, similar to the method applied by Sherif et al. [43], was used in this study, and the supervirtual seismic refraction interferometry (SVI) technique [44,45] was used for part of the SR data, having low signal-to-noise ratio (SNR). After that, the local and global optimization algorithms were applied.

### 2.3. Optimization Principle

#### 2.3.1. Local Optimization Principle

The forward problem in a geophysical inversion is usually solved to describe the relationship between measured data and theoretical data before considering the inverse problem [10]. The solution to the forward problem includes theoretical computation of model response to the earth perturbation, with the assumption of known model parameters and source and receiver positions related to measured data. The field observations can be described as a function of the model parameters due to earth perturbation, plus a certain amount of noise as shown in Equation (1).

(1) $$F\left(m\right)=d_{obs}+n,$$

*F* is the function that describes the sensitivity of the earth model to the earth perturbation, *m* is the model parameter or physical property of the earth, and *n* is noise due to the field survey process. Because the forward operator *F* can be generally expressed as a differential equation, we often solve the forward problem using numerical solution methods such as FD, FE, and IE. 

We aim to estimate the model parameters that produced the noisy field observations in the inverse process. This task is more complicated than the forward calculations because the field observation is sparse relative to the size of the earth model, making the available resources (data) insufficient. Any solution obtained with these limited resources is non-unique; consequently, we add adequate a priori information about the model and the field observations to arrive at the best solution. One such important information source is the data covariance; the theoretical data should vary similarly to the field observations. Additionally, we impose some constraints or apply a smoothness function to the model to obtain a more meaningful geologic interpretation. Generally, we formulate the cost function (*Φ*) by algebraically summing up the data residual and smoothness terms as:

(2) $$\Phi \left(m_{p}\right)=R\left(m_{p}\right)+\alpha_{p}D\left(m_{p}\right),$$

where *R* is the data misfit term, *D* is the stabilizer term which is controlled the smoothness or regularization of model parameters, while *α_p_* is a regularization parameter to balance the weights between data misfit term and stabilizer term, and *m_p_* is a vector representing the model parameter. Specifically, we present the cost function for individual DCR and SR methods as:

(3) $$\Phi \left(m_{dcr}\right)=∥W_{dcr}{\left(d_{dcr}-g_{dcr}\Delta m_{dcr}\right)∥}^{2}+\alpha_{dc}{∥\left(\nabla^{2}m_{dcr}\right)∥}^{2},$$

(4) $$\Phi \left(m_{sr}\right)=∥W_{sr}{\left(d_{sr}-g_{sr}\Delta m_{sr}\right)∥}^{2}+\alpha_{sc}{∥\left(\nabla^{2}m_{sr}\right)∥}^{2}.$$

In Equations (3) and (4), *W* is a weight matrix used for biasing the data, *d* is the field observations, *g* is the Jacobean or sensitivity matrix that is used to obtain the theoretical responses at every model perturbation, ∆*m_p_* is the model correction vector, and ∇^2^ is the Laplacian operator. We minimized the objective function by taking the derivative of the model parameter. Thereafter, the model parameter correction vector for both DCR and SR can be represented as:

(5) $$\Delta m_{dcr}={\left(g_{dcr}^{T}W_{dcr}^{T}W_{dcr}g_{dcr}+\alpha_{dcr}C^{T}C\right)}^{-1}\left(g_{dcr}^{T}W_{dcr}^{T}W_{dcr}\Delta d_{dcr}-\alpha_{dcr}C^{T}Cm_{dcr}^{i-1}\right),$$

(6) $$\Delta m_{sr}={\left(g_{sr}^{T}W_{sr}^{T}W_{sr}g_{sr}+\alpha_{sr}C^{T}C\right)}^{-1}\left(g_{sr}^{T}W_{sr}^{T}W_{sr}\Delta d_{sr}-\alpha_{sr}C^{T}Cm_{sr}^{i-1}\right),$$

where *C* is called as smoothing matrix and includes and includes the Laplacian of the model parameters. Equations (5) and (6) can be simplified as follows:

(7) $$\Delta m_{dcr}=G_{dcr}^{-1}n_{dcr},$$

(8) $$\Delta m_{sr}=G_{sr}^{-1}n_{sr}.$$

We apply the Gauss–Newton optimization algorithm to estimate the model parameter correction vector, and cooling approximation is used to estimate the regularization parameters [46]. Regarding the joint inversion approach, the cross-gradient (structural) constraint is applied as suggested by Gallardo and Meju [11] and Ismail et al. [16,17]. The cross-gradient algorithm considers that the gradient of the model parameters in the joint inversion must be parallel, non-parallel, or equal to zero, which are essential criteria required to satisfy the algorithm. This cross-gradient function for the joint inversion of DCR and SR is given as:

(9) $$\partial \left(m_{dcr},m_{sr}\right)=\nabla m_{dcr}\times \nabla m_{sr},$$

(10) $$≅\partial \left(m_{0dcr},m_{0sr}\right)+B\left(\begin{array}{c} m_{dcr}-m_{0dcr}\\ m_{sr}-m_{0sr} \end{array}\right),$$

where *∂* is the cross-gradient function for all the pixels in the model, m represents the model parameters in both DCR and SR methods, and *B* is the vector of the derivates of the cross-gradient with respect to model parameters. Therefore, the cost function for the joint inversion is formulated as:

(11) $$\Phi \left(m_{dcr},m_{sr}\right)=R\left(m_{dcr},m_{sr}\right)+\alpha_{p}D\left(m_{dcr},m_{sr}\right)+\varepsilon \partial \left(m_{dcr},m_{sr}\right),$$

subject to $\Phi \left(m_{dcr},m_{sr}\right)=0$. In Equation (11), the symbol ‘*ε*’ is used to represent the regularization parameter of the cross-gradient function. The data residual and smoothening terms can be represented as:

(12) $$R\left(m_{dcr},m_{sr}\right)=∥W_{dcr}{\left(d_{dcr}-g_{dcr}\Delta m_{dcr}\right)∥}^{2}+∥W_{sr}{\left(d_{sr}-g_{sr}\Delta m_{sr}\right)∥}^{2},$$

(13) $$D\left(m_{dcr},m_{Sr}\right)={∥\left(\nabla^{2}m_{dcr}+\nabla^{2}m_{sr}\right)∥}^{2}.$$

After performing the minimization of the cost function in Equation (11) as expressed in Demirci et al. [16], the model parameter vector can be represented as:

(14) $$\Delta m=G^{-1}nG^{-1}B^{T}{\left(BG^{-1}B^{T}\right)}^{-1}\left[BG^{-1}n-B\Delta m_{i-1}+\partial \left(m_{i-1}\right)\right].$$

Moreover, the variables ∆*m*, *G*, and *n* in Equation (14) can be expressed in matrix form as:

(15) $$\Delta m=\left[\begin{array}{c} \Delta m_{dcr}\\ \Delta m_{sr} \end{array}\right],G\left[\begin{array}{cc} G_{dcr} & 0\\ 0 & G_{sr} \end{array}\right],\;while\;n=\left[\begin{array}{c} n_{dcr}\\ n_{sr} \end{array}\right].$$

Similarly, to the individual local optimization algorithm, we apply the Gauss–Newton optimization algorithm to estimate the model parameter correction vector, and cooling approximation is used to estimate the regularization parameters.

#### 2.3.2. Global Optimization Principle

Generally, five (5) global optimization algorithms can be used in geophysical inversion [35]; however, the genetic algorithm was applied in this study due to its computational simplicity and it can be easily adapted to parallel computing [35]. Compared with other stochastic methods applied in 1D full waveform inversion, the genetic algorithm provides optimal performance [47]. Fundamentally, the genetic algorithm uses the evolutionary operators selection, crossover, and mutation to compute the solution of an optimization problem [30]. Because the genetic algorithm involves population-based optimization, it is initialized by creating subsets of all the possible solutions in relation to the fitness function of the inverse problem. To obtain the optimality of the global optimization algorithm, both single and multi-objective genetic algorithms were applied. The single objective genetic algorithm involves one fitness function, i.e., only DCR or SR optimization can be run at a time. The binary coding scheme is commonly used to create the initial population for the single objective optimization [35]. Thereafter, genetic algorithm operators are used to search for an optimum solution from the population; this process could be referred to as the stochastic optimization approach. 

The multi-objective genetic algorithm is similar to the single objective; however, instead of coding its population, the model parameters are used directly to initialize the population. In addition, the fitness function is created such that the entire misfit is partitioned according to the number of objective functions, in this case two (i.e., DCR and SR methods). Finally, each portion of the misfit is evaluated with respect to the corresponding fitness functions described in Equations (16) and (17).

(16) $$M_{dcr}=\frac{100\times \left(∥d_{dcr}-td_{dcr}{∥}_{2}\right)}{∥d_{dcr}{∥}_{2}},$$

(17) $$M_{sr}=\frac{100\times \left(∥d_{sr}-td_{sr}{∥}_{2}\right)}{∥d_{sr}{∥}_{2}},$$

where *d* is the field observation (real data), while *td* is the theoretical data for both the DCR and SR methods. The misfit from both DCR and SR objective functions are combined in the multi-objective optimization as:

(18) $$M_{dcr\;sr}=\left[\begin{array}{cc} M_{dcr} & M_{sr} \end{array}\right].$$

To perform the multi-objective optimization in a way that the contribution of each misfit function must be optimized to avoid one method dominating the other (i.e., solutions in the DCR and SR objectives domain are not better than each other), the non-dominating sorting genetic algorithm (NSGA II) is applied here. The concept of this algorithm is shown in Appendix A and discussed explicitly in Deb et al. [48], and has been applied in some geophysical-related studies [29,30,36]. The NSGA II the basic genetic algorithm operators (i.e., selection crossover, and mutation) were used to to create off springs from the initial population and combine both parents and offspring in non-dominated sorting. At the initial stage in this approach, some sets of optimal solutions (non-dominated solutions) arrive at a common front known as Pareto optimal front. These solutions are momentarily set aside in the algorithm while other sets of solutions improve and become non-dominated. Thereafter the principle of elitism [49] is applied to all the non-dominated solutions to determine the sets of optimal solutions that go to the next generation. The combined optimization algorithms involve the application of the output (model) of the local optimization to define a search space (lower and upper bounds) for the global optimization algorithm. A detailed description of the combined optimization algorithm is presented in Edigbue et al. [50].

## 3. Results

### 3.1. Profile 1

**Figure 2:**
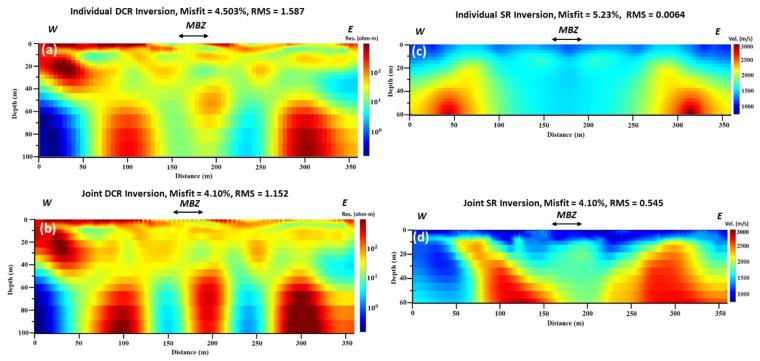
DCR and SR inversion using local optimization method: (**a**) individual inverted resistivity model; (**b**) joint inverted resistivity model; (**c**) individual inverted velocity model; and (**d**) joint inverted velocity model. All geophysical models are vertically exaggerated by 1.35.

The local optimization results for the individual and joint inversions of both DCR and SR are presented in Figure 2. The resistivity model shows that the DCR method penetrates at a depth of 100 m, while the SR method reached the maximum depth of 60 m. It should be mentioned that we decided not to trim the DCR1 data at 60 m depth to be directly comparable with the SR1, since detecting deep structures was one of this study’s objectives. Despite the disparity in depth, both methods show some similarities in the delineating features. The SR method did not significantly trace some of the shallow anomalies, even after applying the joint inversion; this was unlike the DCR method, probably due to the resistivity contrast of these anomalies with respect to the surrounding rock materials. Specifically, surface stratigraphic characteristics, such as the smooth dipping of the layers of the Dammam formation to the east, were detected only on the final inverted individual DCR model. It should be mentioned that the vertical (depth) axis in all final tomographic models is exaggerated by 1.35. Thus, the slope of the layers is much smoother than it appears in the models.

Moreover, low (around 1 Ohm.m.) resistivity vertical structures were depicted at the 150 m and 240 m offset at the DCR final model. From the individual inversion of the seismic refraction data, only the high-velocity structures at the beginning and the end of the model (the bedrock of the study area) are shown, and a wide (more than 100 m thick, from 120–240 m along the SR1 profile), low-velocity zone was presented. However, after applying the joint inversion of the DC (DCR1) and Seismic (SR1) data, the major anomalies were reconstructed with higher resolution. This could be due to the impact of the structural constraint that is applied in the joint inversion. Generally, the DCR and SR data’s joint inversion shows improved structural similarity compared to their individual inversions. Specifically, after applying the joint inversion and using structural constraints, the vertical high resistivity and the in-between low resistivity structure are better depicted, and the shallow structures seem more continuous. The exposed deformed (MBZ) zone and its extent in depth are better seen in the seismic model. In all cases, the final misfit and RMS error are very low, varying from 4.1–5.2% and 0.006–1.6, respectively. 

**Figure 3:**
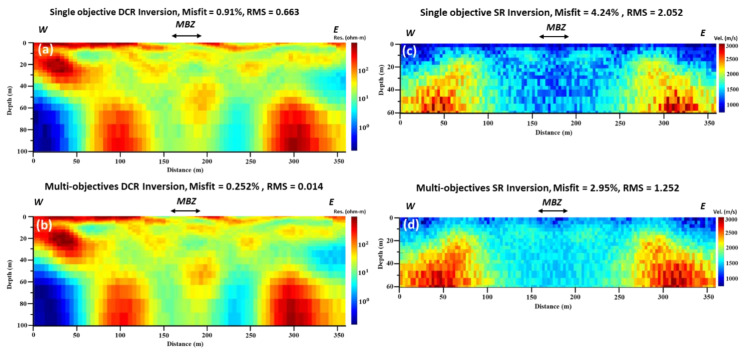
DCR and SR inversion using global optimization method: (**a**) single objective optimization resistivity model; (**b**) multi-objectives optimization resistivity model; (**c**) single objective optimization velocity model; and (**d**) multi-objective optimization velocity model. All geophysical models are vertically exaggerated by 1.35.

The final tomographic models using the global optimization results and applying both single and multi-objective algorithms are presented in Figure 3. The outputs of the single objective global optimization for both data sets (Figure 3, upper row) are structurally similar to the local optimization results. This was partially expected since the search space for the next generation was fed by the output of the local optimization algorithm. At best, it shows a slight improvement in the resolution of the DCR and SR models and significantly decreases the misfit and RMS errors. Minor changes and improvements in the final models were observed by using the multi-objective optimization. Only the misfit and RMS errors are lower. The same resistivity and velocity features were depicted. 

### 3.2. Profile 2

**Figure 4:**
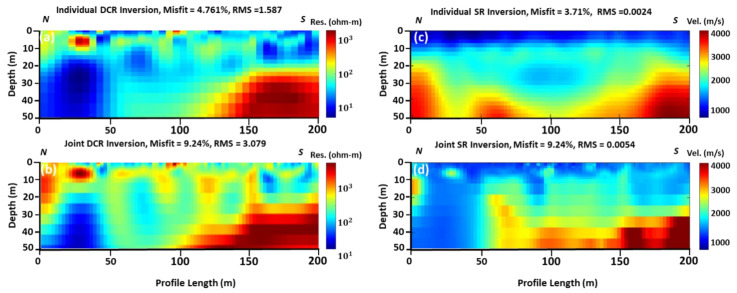
DCR2 and SR2 (see Figure 1b for location) inversion using local optimization method: (**a**) individual inverted resistivity model; (**b**) joint inverted resistivity model; (**c**) individual inverted velocity model; and (**d**) joint inverted velocity model. All geophysical models are vertically exaggerated by 1.35.

Both DCR2 and SR2 data were collected along the same profile. The final tomographic results show that the total penetration depth is 50 m for both the applied techniques. Figure 4, (first column, a and b) shows the results of inversion using a local optimization algorithm for individual and joint inversion of both (DCR and SR) geophysical data. Both tomographic models found the bedrock (high resistivity) formations in the southern part of the acquired profile at 20 m below the ground surface. From the final individual/joint inversion DCR models, the bedrock disappears at around 120 m offset from the north (beginning of the profile). A medium resistivity (100 Ohm.m) formation covers the whole area. In the first 50 m of the profile, and from the 20 m depth to the bottom of the model, a very low resistivity (10 Ohm.m) layer was detected. The final misfit and RMS errors for the final resistivity models are low, varying from 4.76–9.24% and 1.59–3.08, respectively.

The seismic tomographic model of Figure 4c shows a 3-layers velocity model: The upper first layer with an average velocity and thickness of 1000 m/s and 10 m, respectively. The second layer, with an average velocity of 1700–2800 m/s, overlays unconformably the bedrock of the study area, which has an average velocity of 2800–4000 m/s. Figure 4d is affected by the joint inversion and incorporation of the structural constraints of the resistivity model/changes to the final resulted velocity model. A low-velocity anomaly at the first 50 m of the profile was reconstructed, similar to the low-resistivity anomaly detected at the same location from the DCR individual and joint inversion. The final misfit and RMS errors for the final velocity tomographic models are acceptable, varying from 3.71–9.24% and 0.002–0.005, respectively.

**Figure 5:**
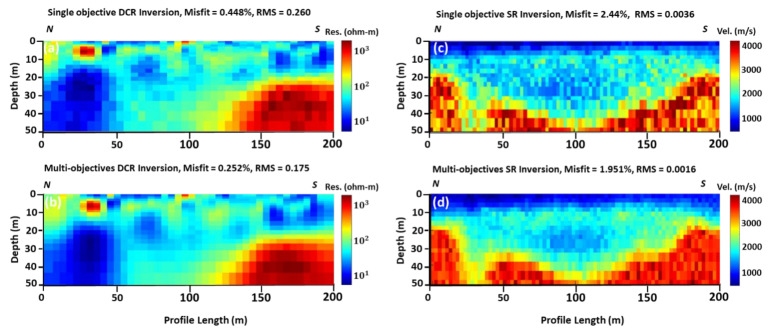
DCR and SR inversion using global optimization method: (**a**) single objective inverted resistivity model; (**b**) multi-objectives inverted resistivity model; (**c**) single objective inverted velocity model; and (**d**) multi-objective inverted velocity model. All geophysical models are vertically exaggerated by 1.35.

Results in Figure 5 show that the global optimization algorithm slightly improves the output of the local optimization algorithm (individual inversion). For instance, in the local optimization algorithm, RMS in DCR2 is between 1.578 and 3.079, while the RMS is between 0.260 and 0.175 in the global optimization results. Likewise, in the case of SR2, RMS is between 0.0024 and 0.0054 in the local optimization result, while RMS is 0.0036 and 0.0016 in the global optimization results. Structurally, the right part of the resistivity model is similar to the right part of the velocity model. The main difference between the two different solutions, using the local and global optimization techniques, can be identified in the models in Figure 4d and Figure 5d. It seems that the structural constraints of the resistivity models from the use of local optimization are not affected in the same way as the models in the global optimization. Thus, Figure 5d shows a similar model as in Figure 4c and Figure 5c, and only a small lateral discontinuity at around 30 m offset is observed, which is probably in the order of the accuracy/resolution of our model at that depth.

## 4. Discussion

The application of the local and global optimization methods was proven promising by using a joint geophysical inversion and interpretation of acquired resistivity and seismic data along an open geological section with visible stratigraphy and tectonic features, where the roadcut was used for ground truthing. The exposed ‘megabreccia’ zone (MBZ), the fault zone within the Dammam formation deposits, was confirmed by both the geophysical (DC and Seismic) data but with different resolutions, as expected based on the advantages and disadvantages of the applied geophysical methods and acquisition protocols used. Based on the composition of the Dammam formation, a low to medium (from 10–100 Ohm.m) resistivity is expected. The high resistivity (more than 100 Ohm.m) and velocity (greater than 2500 m/s) structures at both (west and east) sides of the final geophysical (velocity and resistivity) models can be associated with the Rus formation as mapped by Tleel [7] (Figure 1b), and also reported by Alkhalifa and Kurison [42]. Specifically, to the west of the study area the Rus formation exists in the area, as mapped by Tleel [7], at a distance less than 500 m from the study area.

**Figure 6:**
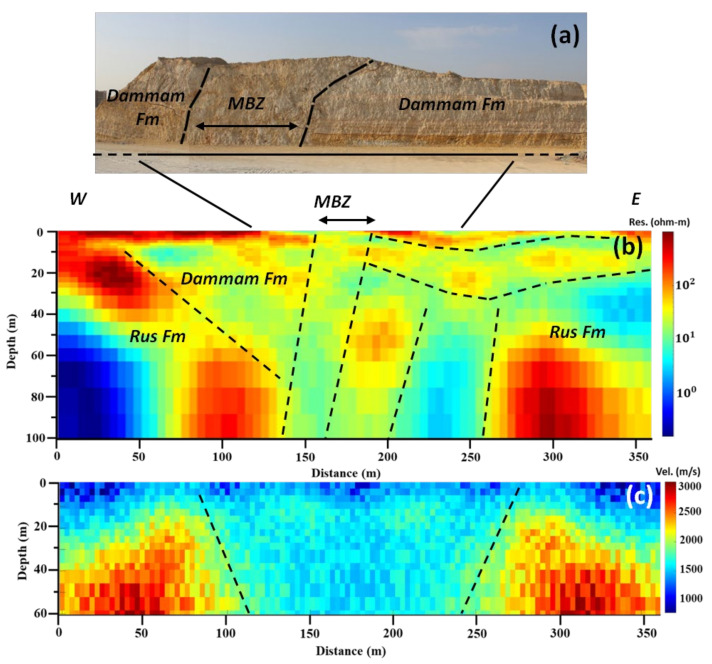
Structural interpretation of the resistivity and velocity models from profile 1: (**a**) section of the outcrop (road-cut) showing inclined strata; (**b**) interpreted resistivity model; and (**c**) interpreted velocity model. Notice that both methods (DCR and SR) map similar structures, especially the faults and the deformed zone. Both geophysical models are vertically exaggerated by 1.35.

In particular, DCR1 profile (Figure 6b) shows in depth the continuation of the MBZ zone, but also one more deformed zone was detected between 200–240 m along the profile with the same low resistivity characteristics. Shallow horizontal to sub-horizontal, medium to high resistivity (50–80 Ohm.m) layers are shown with dashed lines in the eastern part of the profile, which agree with the stratigraphy as observed and is shown in Figure 6a. The final resulting velocity (RS1) model has a much lower resolution and can only image the high-velocity Rus formations at the sides of the model and, with low resolution, the wide deformed (low velocity) zone from 110–250 m along the profile.

**Figure 7:**
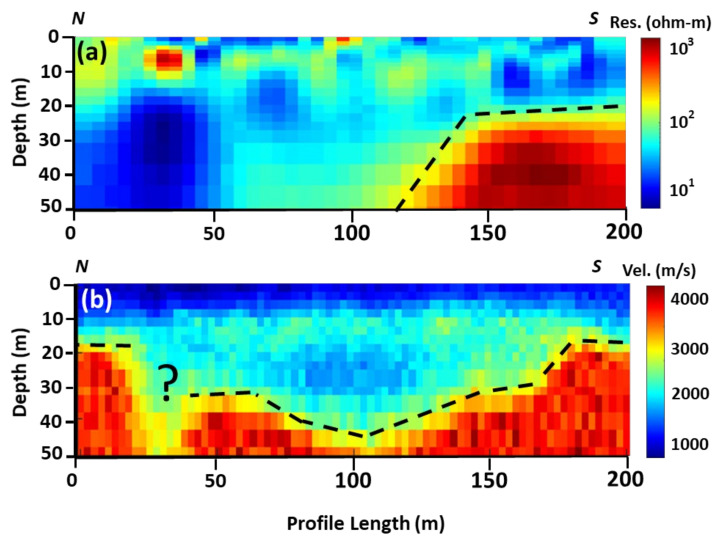
Structural interpretation of the resistivity and velocity models from profile 2 (**a**) interpreted resistivity model; and (**b**) interpreted velocity model.

Although no prominent outcrop reveals structural information on profile 2, the geophysical results show that the method delineated similar major anomalies. Both DCR and SR models in Figure 7 show the bedrock (resistivity of more than 200 Ohm.m and velocity more than 3000 m/s) in the southern part of the profile at a depth of 20 m below the ground surface. The main difference in the interpretation of the different datasets comes from the northern part of the profile. DCR2 shows a clear low (10 Ohm.m) resistivity anomaly, from top to the bottom of the model. In contrast, the SR2 shows the bedrock along the whole profile (dashed line), with a lateral discontinuity (depicted as a question mark in Figure 7b) at the offset of 35–40 m along the profile SR1. This lateral discontinuity, which in applied geophysics can be considered a fracture/deformed zone, can probably be associated with the very low (10 Ohm.m) resistivity anomaly at the same offset. Since profile 2 has an almost N–S direction, it can be safely assumed that the low detected resistivity and probably lower velocity anomalies are related to a deformed and/or fractured zone of the WSW–ENE direction. This finding agrees with the direction of the subsurface fractures reported by Chavanidis et al. [44], supporting the conclusion that deep and thicker fractured/deformed zones of the WSW–ENE trend, perpendicular to the main axis of the Dammam Dome, exist. 

## 5. Conclusions

Both local and global optimization algorithms were applied individually and jointly to achieve the high-resolution resistivity and velocity models presented in this study. The local optimization results show satisfactory outputs as a result of using appropriate regularization parameters for both DCR and SR data inversion. In the combined optimization algorithm, results from the individual local optimization algorithm were used as input to define the search space for the global optimization method. Since the local optimization algorithm performs acceptably, the global optimization method slightly improves the model resolution and RMS error. Subsurface deformational features such as fractures/faults were mapped on both profiles in the study area. Profile 1 reveals significant NNW–SSE faults, as lateral discontinuity of the resulting tomographic models, observed in both the resistivity and velocity models. Moreover, the observed surface MBZ along the road-cut was confirmed in-depth. The observed anomalies from both resistivity and velocity models for profile 2 show similarities to those in profile 1. Specifically, the interpretation of profile 2 has identified low resistivity and low-velocity anomalies which can be safely related to a deformed zone of WSW–ENE direction. Similar in-trend, subsurface fractures were reported by Chavanidis et al. [44]. It is shown that data integration, or joint geophysical data inversion using the combined local and global optimization methods, can unravel the geology of complex areas in depth. Integrating both DCR and SR data increases the model resolution and provides well-correlated structural similarities in the models. Moreover, these results show the feasibility of applying the combined local and global optimization algorithm to jointly interpret real 2D DCR and SR data, using both single and multi-objective genetic algorithm methods. Although the strength of the genetic algorithm lies in simplicity and adaptability to parallel computers, however, there is the tendency for early convergence (weakness), especially when there is over-mutation and crossover. Future studies may focus on determining the genetic algorithm optimum parameters (i.e., mutation rate and type of crossover) for the inversion of large datasets (e.g., 2D DCR data).

## Data Availability

Not applicable.

## Appendix A. A Template of the Combined Application in Seismic and Geoelectrical Tomograhy

% Input geometrical parameters of 2D model and initial model velocities/resistivities:

% X-limits of model: xmin, xmax

% Node spacing in x and z directions: dx, dz

% Max depth of model: zmax

% Initial velocit model: v

fid=fopen(‘InitModel.md’,‘r’);

[xmin, xmax, dx, dz, zmax,v]=finput(fid);

xnodes=((xmax-xmin)/dx)+1; % Number of nodes along x- direction

znodes=((zmax-zmin)/dz)+1; % Number of nodes along z- direction

nparam=xnodes*znodes; % Number of unknown parameters

%Input measured data (traveltimes, resistivities)

load SR.dat

load Res.dat

%% Local optimization section

% Insert the fittest model as initial model for inversion

  niter=10;%number of iterations

  [RMS,mnew]=INVgen(xmin,zmin,dx,dz,xmax,zmax,v,idealtt,niter);

[RMS,mnew]=INVgen(xmin,zmin,dx,dz,xmax,zmax,rho,app_rho,niter);

bestfit=RMS;

    if RMS<bestfit

    arent(loc,:)=mnew1;

    arent(loc,:)=mnew2;

    end

%% Global optimization section

%Define parameter search spaces for GA by giving a %15 variance to initial values

% define the search space for the global optimization

v=mnew1

rho=mnew2

parmin(1:nparam)=v/1.15; % lower bound of the search space

parmax(1:nparam)=v*1.15; % lower bound of the search space

  %GA parameter definitions

microaga=1; % use of micropopulation instead of mutation

pcross=0.65; % crossover probability

pmutate=0.02; % mutation probability if (microga turned off)

maxgen=30; % number of generations

npopsiz=10*nparam; % An empirical value for the population size

  % Generate the initial population randomly

[iparent]=initial(nparam,parmin,parmax,npopsiz);

% Main evolutionary loop

for igen=1:maxgen

% Decode the binary coded strings into decimal

  parents=decode(iparent);

  if npopsiz>100

% Run forward calculations on local machine

    ttminback=refraction(igen,xmin,zmin,dx,dz,xmax,zmax,parents); % vectorized

    res=dcr(igen,xmin,zmin,dx,dz,xmax,zmax,parents); % vectorized

  else

% Distribute the calculations over the cpu’s

    jm = findResource(‘scheduler’,‘type’,‘jobmanager’,‘LookupURL’,‘HOSTNAME’);

    job1 = createJob(jm);

% SR.dat: Sources - Receivers location

% refraction.m: an algorithm to solve the forward problem

    set(job1,‘FileDependencies’,{‘refraction.m’,‘SR.dat’});

    dl=1; % down limit

    groupsize=npopsiz/(jm.NumberOfIdleWorkers);

    ul=groupsize; % upper limit

    NumberofTasks=npopsiz/groupsize;

    for itask=1:NumberofTasks

createTask(job1,@refraction,1,{igen,groupsize,xmin,zmin,dx,dz,xmax,zmax,parent(dl:ul,:)});

      dl=dl+groupsize;

      ul=ul+groupsize;

   end

   submit(job1)

   waitForState(job1,‘finished’);

   out=getAllOutputArguments(job1);

   ttminback=cell2mat(out(:,1)‘)’;

   finished_jobs = findJob(jm,‘State’,‘finished’);

   destroy(finished_jobs)

  end

% Compute measured-calculated traveltimes misfit by means of RMS

  load measured_tt.dat;

  misfit=norm(measured_tt-ttminback)/sqrt(length(ttminback));

  average_misfit(igen)=sum(misfit)/npopsiz;

  [bestfit,loc]=min(misfit); %best fitted individual

  fittestvel=parrent(loc,:);

% Compute measured-calculated traveltimes misfit by means of RMS

  load measured_res.dat;

  misfit=norm(measured_res-res)/sqrt(length(res));

  average_misfit(igen)=sum(misfit)/npopsiz;

  [bestfit,loc]=min(misfit); %best fitted individual

  fittestrho=parrent(loc,:);

% Define the fittest velocity models compared to the average misfit

   [ix]=find(misfit < (average_misfit(igen)));

   nmoditer=length(ix);

   for ii=1:nmoditer;

% Run the forward modeling once more for each selected vel model

% To estimate the Jacobian (derivative) matrix

      bestvel=parent(ix(ii),:);

      ttminback=refraction(igen,1,xmin,zmin,dx,dz,xmax,zmax,bestvel);

      res=dcr(igen,1,xmin,zmin,dx,dz,xmax,zmax,bestrho);

      new_min=max(mnew);

      new_max=min(mnew);

      save (‘parmin.mat’,‘new_max’,‘new_min’);

% The MNEW is the updated velocity model and transformed to

% Binary format to include these best vel models to the original population

       if RMS<misfit(ix(ii))

       binarray=code2(nparam,mnew,ig2,g0,g1);

       iparent(:,ix(ii))=binarray;

       misfit(ix(ii))=RMS;

       end

     end; % End of the inversion of all the fittest models

  end;

% End of the check about the partial inversion before continue the gen

% Selection crossover and mutation

  mate1=selecter(misfit,npopsiz);

  mate2=selecter(misfit,npopsiz);

  ichild=iparent(n,mate1);

  ras=rand(1,nchrome);

  mer=find(ras<=pcross);

  ichild(mer,1:npopsiz)=iparent(mer,mate2);

% End of evolution

% Code the new generation

  iparent=newgen(ielite,npossum,ig2sum,ibest,npopsiz,ichild,iparent);

  if microga==1;

    [iparent]=gamicro(npopsiz,nchrome,iparent,ibest);

  end

    if best<1e-09

    break

  end

%    Save results

    string_gen=int2str(igen);

  save_file=strcat(‘Results’,string_gen,‘.mat’);

  save(save_file, ‘parents’, ‘xp’, ‘zp’, ‘bf’, ‘average_misfit’,‘misfit’);

end % Close the iteration over all generations

fclose(‘all’);

% End of Code
